# An umbrella review of health outcomes following traumatic brain injury

**DOI:** 10.1038/s44220-024-00356-5

**Published:** 2025-01-03

**Authors:** Maya G. T. Ogonah, Stella Botchway, Rongqin Yu, Peter W. Schofield, Seena Fazel

**Affiliations:** 1 Department of Psychiatry, University of Oxford, Warneford Hospital, Oxford, UK; 2 School of Medicine and Public Health, University of Newcastle, Newcastle, New South Wales Australia; 3 Neuropsychiatry Service, Hunter New England Local Health District, Newcastle, New South Wales Australia

**Keywords:** Brain injuries, Public health, Psychology

## Abstract

While numerous reviews have assessed the association between traumatic brain injury (TBI) and various mental and physical health outcomes, a comprehensive evaluation of the scope, validity, and quality of evidence is lacking. Here we present an umbrella review of a wide range of health outcomes following TBI and outline outcome risks across subpopulations. On 17 May 2023, we searched Embase, Medline, Global Health, PsycINFO, and Cochrane Database of Systematic Reviews for systematic reviews and meta-analyses. We compared risk ratios across different outcomes for risks compared with people without TBI and examined study quality, including heterogeneity, publication bias, and prediction intervals. The study was registered with PROSPERO (CRD42023432255). We identified 24 systematic reviews and meta-analyses covering 24 health outcomes in 31,397,958 participants. The current evidence base indicates an increased risk of multiple mental and physical health outcomes, including psychotic disorders, attention-deficit/hyperactivity disorder, suicide, and depression. Three outcomes—dementia, violence perpetration, and amyotrophic lateral sclerosis—had meta-analytical evidence of at least moderate quality, which suggest targets for more personalized assessment. Health-care services should review how to prevent adverse long-term outcomes in TBI.

## Abstract (web summary)

This umbrella review synthesizes a large body of evidence on adverse outcomes in over 31 million people with traumatic brain injury and identifies links with dementia, perpetration of violence, and amyotrophic lateral sclerosis.

## Main

Traumatic brain injury (TBI) is a global public health concern. Incidence rate for TBI is estimated internationally at 349 per 100,000 person-years,^1^ and it is predicted that nearly 50% of the global population will sustain a TBI during their lifetime.^2^ TBI has the highest incidence and prevalence of all common neurological disorders.^3^ Studies have reported associations between TBI and many health outcomes, including neurodegenerative diseases,^4–6^ cognitive impairment,^7^ stroke,^8–10^ psychiatric illness,^11,12^ and mortality.^13,14^ However, previous work to quantify links between TBI and health outcomes has focused on single conditions or disease groups, with results not systematically summarized across all health domains. This makes appraisal of evidence difficult, and the overall public health impact of TBI is likely underestimated. In addition, estimates of the magnitude of the strength of associations between TBI and subsequent health outcomes have been inconsistent and may be prone to bias. Thus, in this umbrella review, we aim to synthesize research on potential sequelae of TBI, identify evidence gaps, and systematically assess study quality and bias.^15^ This is particularly relevant as the global incidence of TBI is rising, in part due to a notable increase in reported trauma-related violence and road traffic accidents in low- and middle-income countries.^16–18^

This synthesis can help quantify the overall disability burden from TBI and inform targets for clinical and policy interventions. We also aim to summarize outcome incidence after TBI across vulnerable subpopulations,^3,19^ and the effects of TBI severity. Findings could guide appropriate allocation of health-care resources, prognostic assessment, and implementation of interventions.

## Results

### Study selection

**Fig. 1:**
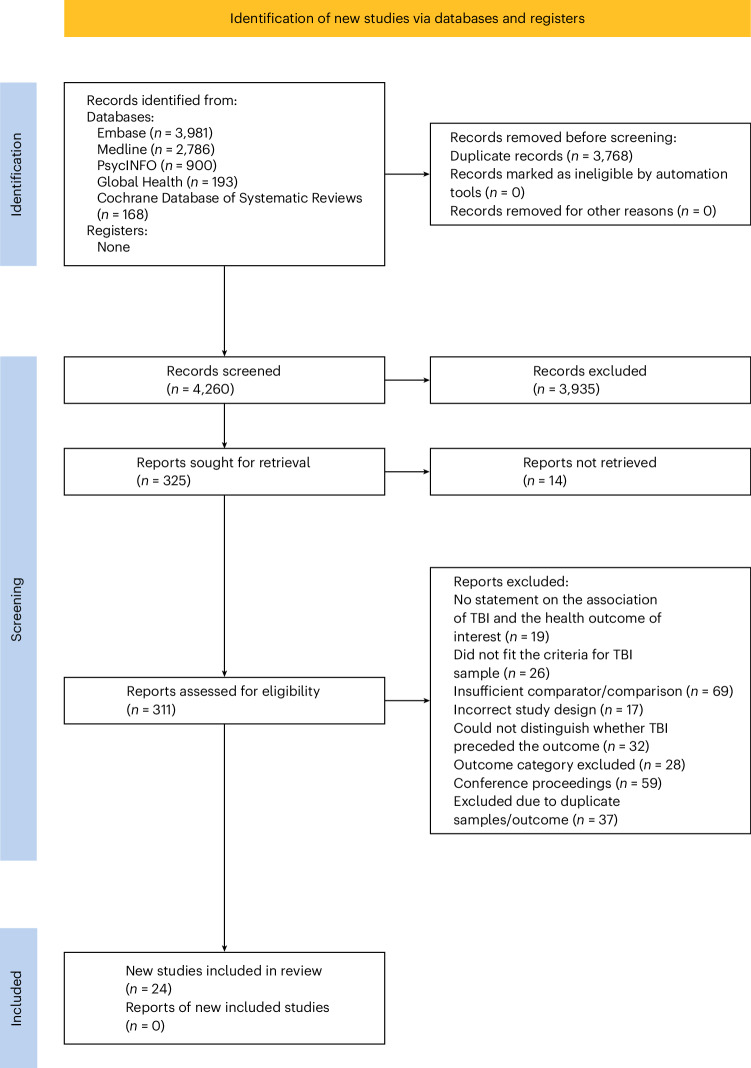
PRISMA flow diagram. Preferred Reporting Items for Systematic Reviews and Meta-analyses (PRISMA) flow diagram.

The database and manual search yielded 8,028 articles. After de-duplication, 4,260 were assessed at title and abstract screening (Fig. 1). In total, 325 studies were screened for full text, and 24 systematic reviews and meta-analyses were deemed eligible, covering 24 mental and physical health outcomes (Supplementary Table 3).

### Study characteristics

Of the 24 articles identified, 17 had a quantitative synthesis providing a summary estimate. All reviews were published between 2009 and 2023, with primary studies from 1952 to 2022. These investigations included a total of 31,397,958 individuals exposed and unexposed to TBI, with participant numbers in each primary study ranging from 338 to 9,861,153. For results synthesis, TBI was stratified into mild (including concussions), moderate, and severe TBI categories where possible, in line with current research. Due to insufficient reporting of injury characteristics in most reviews, we relied on each review’s definitions of mild, moderate, and severe TBI, which varied across them. When studies did not specify or stratify by severity of exposure, TBI was collapsed into a general TBI of any severity category. A minority of studies focused on the effect of TBI on health outcomes in vulnerable subpopulations, with five studies (two meta-analyses and three reviews) focusing on pediatric and adolescent TBI, four studies (two meta-analysis and two reviews) that included military personnel or veterans, four reports (two meta-analyses and two reviews) focusing on sports-related TBI, and one meta-analysis in older adults.

### Narrative synthesis

The seven systematic reviews without quantitative synthesis examined mortality (number of studies (*k*) = 1),^20^ physical health outcomes, including chronic pain (*k* = 1),^21^ and gait impairment (*k* = 2),^22,23^ and mental health outcomes, including post-traumatic stress disorder (PTSD; *k* = 1),^24^ depression, anxiety, oppositional defiant disorder, autism, and substance misuse (all one study)^25^ following TBI. In these systematic reviews, authors reported some evidence for associations between TBI and impaired gait, chronic pain, PTSD, substance abuse, and increased mortality, on the basis of narrative synthesis. Two reviews did not conduct quantitative synthesis because of large heterogeneity in study populations, research design methodology, and exposure and outcome measurement; five studies did not provide rationale for not completing a meta-analysis.

### Relative risk

**Fig. 2:**
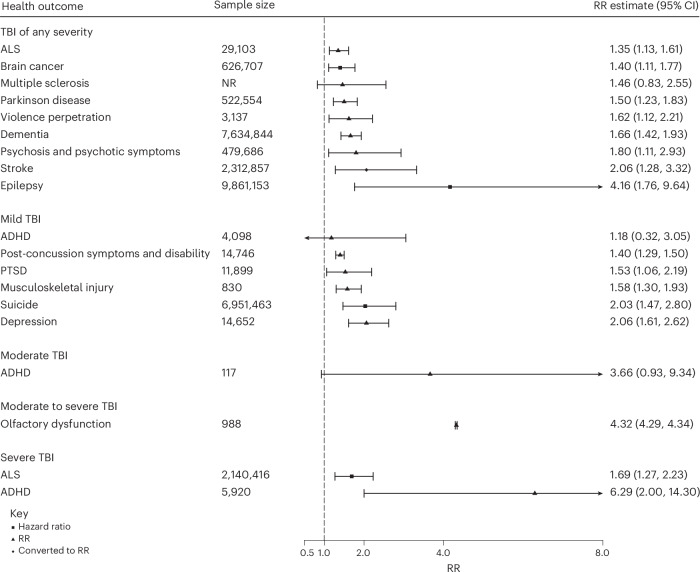
Risk estimates of health outcomes following TBI stratified by injury severity. Data are presented as RR ± CI.

The relative risk (RR) for TBI not stratified by severity ranged from 1.4 (95% confidence interval (CI): 1.1–1.6) for amyotrophic lateral sclerosis (ALS) to 4.2 (1.8–9.6) for epilepsy (Fig. 2). The RR for mild TBI ranged from 1.2 (0.3–3.1) for attention-deficit/hyperactivity disorder (ADHD) to 2.1 (1.6–2.6) for depression. In those with moderate TBI, ADHD was associated with a RR of 3.7 (0.9–9.3). In those with moderate to severe TBI, olfactory dysfunction was associated with a RR of 4.3 (4.3–4.3). The RR for severe TBI was 1.7 (1.3–2.2) for ALS and 6.3 (2.0–14.3) for ADHD.

### Findings by subpopulation

Of the two meta-analyses in pediatric populations, severe TBI was significantly associated with an increased risk of ADHD (RR = 6.3 (2.0–14.3)), and TBI of any severity was associated with psychosis (RR = 1.8 (1.1–2.9)). Evidence from three reviews indicated that, in pediatric populations, PTSD and gait impairment were more common following TBI compared with control groups without TBI. In sports-injured participants, mild TBI was linked with an increased risk of musculoskeletal injury and gait impairment; however, the association between TBI and postural control was not statistically significant. Further, one review^26^ found a similar trend in depression and anxiety symptoms in athletes with sports-related concussions compared with controls. Evidence indicates an increased risk of suicide and dementia following TBI in veterans (although the difference in risk between veterans and civilians was not statistically significant).^27,28^ One review^21^ found that in military or veteran populations, mild TBI was associated with a higher prevalence of chronic pain (compared with non-TBI veterans). Further, moderate to severe TBI was associated with increased mortality in the post-TBI acute phases.^20^ The sole meta-analysis in older adults^29^ indicated a significant link between TBI and Parkinson disease (RR = 1.5 (1.2–1.8)).

### Population-attributable fractions

Assuming causality, population-attributable fractions (PAFs) for TBI not stratified by severity ranged from 4.0% (3.9–4.2%) for ALS (Table 1) to 27.5% (27.5–27.5%) for epilepsy. For mild TBI, PAFs ranged from 2.1% (–3.4% to 7.6%) for ADHD to 11.3% (10.8–11.8%) for depression; for moderate to severe TBI, PAFs ranged from 7.6% (7.1–8.2%) for ALS to 38.8% (38.7–40.0%) for ADHD (Supplementary Table 4 and 5).

**Table 1:** Effect of TBI of any severity on health outcomes and PAFs

Health outcome	Population	RR	95% CI	95% prediction interval	PAF (%)	95% CI
Epilepsy	General	4.16	1.76–9.64	··	27.5	27.5–27.5
Stroke	General	2.06^a^	1.28–3.32^a^	0.53–5.95	11.3	11.2–11.3
Psychosis and psychotic symptoms	Pediatric	1.80	1.11–2.93	0.71–4.54	8.8	8.6–8.9
Dementia	General and military	1.66	1.42–1.93	0.72–3.82	7.3	7.3–7.4
Violence perpetration	General	1.62	1.12–2.21	1.03–2.67	6.9	5.1–8.8
Parkinson Disease	Older adults	1.50	1.23–1.83	0.66–3.40	5.7	5.5–5.8
Multiple sclerosis	General	1.46	0.83–2.55	··	5.2	··
Brain cancer	General	1.40	1.11–1.77	0.64–3.09	4.6	4.4–4.8
ALS	General	1.35	1.13–1.61	0.93–1.96	4.0	3.9–4.2

^a^Hazard ratio is presented.

–, Prediction interval could not be calculated.

### Quality assessment

**Fig. 3:**
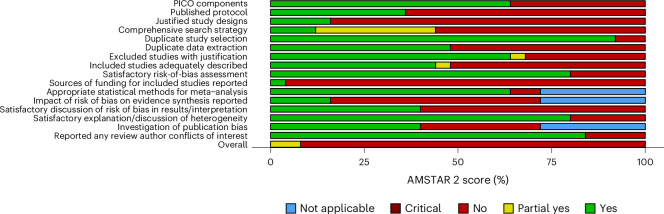
AMSTAR 2 scores across domains. Items covering the appropriateness of the meta-analytical methods (items 11, 12, and 15) were not applicable for systematic reviews. 'PICO' refers to population, intervention, control, and outcomes.

In terms of quality, of the 24 reviews, 2 (8.3%) were moderate, 3 (12.5%) were low, and 19 (79.1%) were critically low according to AMSTAR 2 (ref. ^30^) (A Measurement Tool to Assess Systematic Reviews version 2) criteria. None was rated as high quality. For the seven critical domains, the lowest ratings were: 35% of included reviews explicitly established a protocol before conducting the review, 38% accounted for risk of bias in primary studies when interpreting and discussing results, and 42% used a comprehensive literature search strategy (Fig. 3 for AMSTAR 2 scores across domains and Supplementary Table 6 for AMSTAR 2 ratings for each review).

Eleven meta-analyses assessed publication bias, and five did not exhibit evidence of small-sample bias or asymmetry in funnel plot distribution. Over two-thirds (13/17) of studies had high statistical heterogeneity; however, most (16/17) explored sources of this heterogeneity through subgroup or meta-regression techniques. For 11 meta-analyses, 95% prediction intervals could be calculated, with 4 having intervals excluding the null prediction interval of 1 for the risk developing olfactory dysfunction, post-concussion symptoms, ALS (in cases of severe TBI), and violence perpetration. Of the 17 eligible meta-analyses, 1 (5.9%) meta-analysis was rated high quality, 2 (11.8%) were moderate, and the rest were low quality (83.3%). Supplementary Table 7 provides a full quality assessment for each meta-analysis.

## Discussion

In this comprehensive umbrella review of 24 mental and physical health outcomes following TBI, we have summarized evidence from more than 31 million participants. Overall, TBI was associated with multiple mental and physical adverse health outcomes. Among these, only three were based on high- or moderate-quality meta-analytic evidence: dementia,^27^ violence perpetration,^31^ and ALS.^32^ Violence perpetration and ALS (following severe TBI) also exhibited significant 95% prediction intervals, suggesting that in most patient populations, TBI increases risk of these outcomes.

However, outcomes were typically not stratified by TBI severity. A large proportion of studies did not conduct analyses based on injury severity or specify whether injuries were complicated (for example, with skull fractures, contusions, or hemorrhages). Even within mild TBI, there is a wide range from momentary loss of consciousness to consciousness loss of up to 30 minutes. Although mild TBI is often purely physiological, in a fraction of cases, structural brain pathology can occur,^33^ with consequences for clinical sequelae. Meta-analyses of ADHD risk following TBI found that risk was not significantly increased compared with children of other injuries following mild or moderate TBI, but risk significantly increased following severe TBI,^34^ underscoring the importance of stratification by severity.

Our review has highlighted several research gaps. Future research should seek to account for dose effects of multiple TBIs. Previous research has found differences between groups defined by the number of reported TBIs; multiple recurrent injuries was associated with a greater risk of adverse health outcomes, and lower social support and quality of life.^35^ Limited evidence in this review sought to examine the potential effect of recurrent TBI; one included study^32^ found that recurrent injuries slightly strengthen the association between TBI and ALS; however, wide confidence intervals included null. One review^36^ found that the odds of a neurological or psychiatric diagnosis were not higher for multiple TBIs compared with those with a single injury; however, it was rated low quality, and findings were not specific to one outcome and hence should be interpreted with caution.

Our review sought to investigate the impact of TBI on vulnerable subpopulations, including children and adolescents, sports participants, military personnel and veterans, and older adults; however, no systematic reviews or meta-analyses on health outcomes following TBI in survivors of intimate partner violence (IPV) were identified. Considering the high prevalence of IPV (approximately 30% of women globally have experienced physical or sexual violence),^37,38^ and that survivors of IPV are at high risk of TBIs^39^ (in particular, recurrent TBIs)^40,41^ while often discouraged from seeking medical treatment, further research in this vulnerable population is necessary.

Poor reporting also limited analyses examining the effects of the nature of the control group on findings. Studies that recruit healthy controls may yield larger effect-size estimates than those recruiting orthopedic or other-injury controls, as injured persons also suffer adverse consequences of trauma exposure and bodily injury. Therefore, some of the increased risk post-injury might be due to sustaining an injury, rather than being specific to TBI. Limited evidence sought to compare effect sizes between different types of controls; one study with no clear differences between the risk of ADHD post TBI in non-injured compared with other-injured controls, tested effects of ADHD comorbidity. Furthermore, demographic information was often combined in TBI exposed and non-exposed groups. There may be differences in the baseline characteristics of survivors of TBI compared with controls. Pooling information across groups limits the identification of relevant characteristics that could confound any association.

Through our search strategy, we identified 13 systematic reviews or meta-analyses on risk of dementia following TBI (only one of which was included in this umbrella review due to overlapping samples), most of which were published in the past 5 years. There appears consensus in the literature, with previous meta-analyses on dementia post-TBI reporting pooled risk ratios between 1.6 and 1.9.^4,5,42–46^ Overlapping meta-analyses on the same topic indicate potential redundancy,^47^ which registration of protocols could prevent. We also recommend future meta-analyses report prediction intervals for the summary effect, which only one included meta-analysis^27^ did. Where there is between-study heterogeneity, common for meta-analyses, the prediction interval will cover a wider range than a confidence interval. This was the case in our review; although TBI was associated with all health outcomes investigated, the prediction interval was significant for only four outcomes (post-concussion symptoms, violence perpetration, olfactory dysfunction, and ALS).

Further, future research should account for the impact of time interval on risk. We excluded studies that did not clearly indicate that TBI preceded the health outcome or consisted predominately of cross-sectional studies. This exclusion aimed to minimize the potential bias introduced by preclinical diseases conferring an elevated risk of TBI. Nevertheless, substantial heterogeneity was observed among primary studies and between reviews concerning the time interval between TBI and health outcome. Only six meta-analyses investigated the impact of time interval on risk. One review in college athletes found that the risk of lower musculoskeletal injury was elevated in the first 90 days after return to play, although the link was non-significant after one year.^48^ It is theorized that after sustaining a concussion, athletes may experience neuromuscular control deficits that increase their risk of musculoskeletal injury in the short but not long term.^48^ Risk of epilepsy was highest within the first 5 years post-injury, while attenuated, but still significant, after 5 years.^49^ TBI can alter brain activity, resulting in epilepsy or seizures shortly after injury, but it may also contribute to biological changes that occur over time (such as neurodegeneration, regeneration, and remodeling), resulting in later development of post-traumatic epilepsy. Moreover, research found that the risk of dementia^27^ and ALS^32^ was lower when studies required at least a 1 year time lag, suggesting that the risk of certain adverse outcomes may be elevated shortly after TBI diagnosis. However, this is likely attributable to reverse causation, with head injury being an early sign of prodromal illness, or to medical examinations following TBI revealing pre-existing health conditions with enhanced neurological case finding. Therefore, risk estimates from studies with longer follow-up may be more informative to clinical decision-making, and future research should aim to investigate how the risk of each outcome post-TBI varies over time.

Our findings underscore the need for effective interventions to address risk of longer-term outcomes. Early identification of patients at risk of adverse outcomes is important for guiding optimal management. Enhancing health literacy and awareness through psychoeducation may improve quality of life. For example, increasing awareness of possible violent behavior following brain injuries could lead to risk management, with potential prevention of harms to carers and others. Improved understanding of TBI sequelae can allow for appropriate targeting of health-care resources and implementation of interventions to maintain well-being.^50^ Interventions aimed at preventing recurrent brain injuries, such as more use of child head helmets, are likely to be part of wider public health measures.

A strength of this umbrella review is that it focuses on diagnosed health conditions, rather than on self-report symptoms or patient performance on standardized tests; by focusing on clinical criteria, our findings may better reflect the disease burden and the challenges of disease management. Moreover, temporal ordering between the exposure and outcome of interest reduced the risk of reverse causation. Another strength is our quality assessment procedure, allowing for comparison between higher- and lower-quality evidence findings.

One limitation is that the quality assessment (AMSTAR) tool was developed to evaluate systematic reviews of randomized trials, and the threshold for bias might be too low for reviews of observational studies. We addressed this limitation by including other measures of bias, including prediction intervals, statistical heterogeneity, additional subgroup and meta-regression analyses, and publication bias. Second, we note that the true burden of disease due to TBI may be lower than that reported in this review. Calculating PAFs as described makes assumptions about causality (that is, between exposure and outcome), independence of the risk factors (whereas risk factors likely coexist and interact), and homogeneity of the relative risk across the population. Violation of these assumptions may lead to biased estimates of the PAF; however, due to the limited information available from the systematic reviews, this remained the best approach for our analysis. These results highlight the substantial burden of disease that could be attributable to TBIs. However, we encourage caution in the interpretation of the unadjusted PAFs, which would attenuate with better-quality designs in primary studies, and PAFs assume causality.

## Conclusion

In summary, this umbrella review has shown that TBI is associated with an elevated risk of a range of adverse health outcomes. In particular, there is higher-quality evidence that finds increased risk of dementia, violence, and ALS, following TBI. We highlight the need for better exposure reporting and stratification of individuals by TBI severity. Our findings suggest that TBI may represent an important modifiable risk factor for a range of outcomes, and improving assessment and early intervention may be a focus for future research. Further awareness of the consequences of TBI outside the intensive care and immediate hospital period is needed; TBI should not only be regarded as an acute condition but can be a chronic disease associated with long-term health outcomes, negatively impacting quality of life. Public health and policy awareness of the extent, range, and severity of the consequences caused by TBI can inform service development. Furthermore, health-care services should review their approaches to prevent these longer-term consequences.

### Recommendations for practice

Taken together, the findings provide some potential directions for clinical practice. Individuals with TBI are at risk of a range of adverse outcomes, including those presenting with mild TBI and concussion. Protocols for early treatment of potential long-term problems are necessary to ensure those with TBI are provided with sufficient support and that long-term consequences are mitigated where possible. The review’s findings demonstrate the complex paths and wide range of outcome domains following head injury. In particular, risks of dementia, violence perpetration, and ALS should be considered. Pre-injury factors such as substance misuse and psychiatric conditions may lead to poor prognosis. Precision medicine approaches to TBI management can assist in better outcome prognosis and appropriate targeting of treatments and other health-care resources.

## Methods

The umbrella review was conducted according to a predetermined protocol (CRD42023432255) registered with the International Prospective Register of Systematic Reviews (PROSPERO) on 5 September 2023. It follows JBI methodology^51^ and is reported in compliance with Preferred Reporting Items for Systematic Reviews and Meta-analyses (PRISMA) guidelines^52^ (Supplementary Table 1). JBI methodology for umbrella reviews is a structured approach for synthesizing evidence across multiple systematic reviews. Key elements of JBI umbrella reviews include a comprehensive search strategy, specific inclusion criteria from clearly identifiable PICO, and rigorous assessment of methodological quality.

### Search strategy

We conducted an umbrella review in which information from existing systematic reviews and meta-analyses of studies on health outcomes following TBI was systematically collated and evaluated. We developed a search strategy in consultation with a research librarian. We did a keyword search on four major electronic databases via Ovid—Embase, MEDLINE, Global Health, and PsycINFO—alongside searching Cochrane Database of Systematic Reviews for papers published from database inception until 17 May 2023. No limits or restrictions were used; gray literature was eligible, with no search filters (Supplementary Table 2 provides search terms). Reference lists of retained studies were hand searched to identify additional studies, and gray literature was searched on Google Scholar.

### Eligibility criteria

Eligibility criteria were established using the Population, Intervention/Exposure, Comparators, Outcomes, Timing/Setting (PICOT) framework. The inclusion criteria were (1) study type (systematic reviews and meta-analyses); (2) population (individuals who have experienced TBI, or a comparable head injury, with both self-reported and clinician diagnosis considered and no restriction on the cause and severity of TBI); (3) exposure (TBI, which for this review is defined as any change in brain function resulting from an external force,^53^ such as a blow or injury to the head, accelerating/decelerating movement, and forces generated from blast events); (3) comparator (systematic reviews were eligible only if they included more than one study with control groups (for example, healthy controls, orthopedic controls) or pre-TBI measurements, so that TBI-specific differences could be identified, synthesized across studies, and compared); (4) outcome (adverse health outcomes—physical and mental health); (5) timing (health outcome occurred subsequent to TBI).

Due to differences in pathological mechanisms, brain injury caused by hypoxia–ischemia was excluded. Non-systematic reviews, interventional studies, case studies, observational studies, discussion papers, editorials, letters, and conference abstracts were also excluded. Cognitive and social outcomes were considered outside the scope. Adverse health outcomes refer to detrimental effects on an individual’s physical or mental health, encompassing a wide range of conditions that may contribute to disability, increased morbidity, or decreased quality of life. Reviews that investigated the relationship between TBI and poor functional outcomes/disability (for example, as measured by Glasgow Outcome Scale^54^) were also deemed eligible. However, other disability measures such as return to work post-injury were considered related to social outcomes and thus outside the scope of the current review.

When more than one review reported data for the same exposure–outcome relationship and included the same primary articles published outside a 3 year period, the most recent review that met inclusion criteria was selected, and the older review was excluded to avoid sample duplication. When two or more studies reported data for the same outcome and included the same primary articles and were published within a 3 year period, we selected the review of higher quality (based on quality assessment) and excluded others. The PRISMA2020 package^55^ was used to produce the PRISMA flow diagram.

### Data collection

M.G.T.O. screened the titles and abstracts of the identified studies, with 20% independently double screened by S.B. The percentage agreement between raters at title and abstract stage was 95.5% (Cohen’s *κ* = 0·71), indicating moderate inter-rater reliability.^56^ Percentage agreement at full-text stage was 84.1% (Cohen’s *κ* = 0.66). Any disagreements were resolved via consensus. The articles were managed using Endnote X9^57^ and Rayyan^58^ software.

### Data extraction

Data were extracted using a standardized Excel spreadsheet, which was piloted on five studies. Predetermined study characteristics were extracted, including number of eligible studies, sample size, effect estimates with 95% CIs, and quality assessment. Information on the nature of a study’s control group was extracted, including whether healthy/non-injured controls or other-injured controls were included. When possible, we extracted demographic information for exposed and control groups separately; however, this information was often pooled across groups. For some articles, not all primary studies met inclusion criteria; therefore where possible, data were extracted for the studies/subgroups that met inclusion criteria. Thus, the number of studies or participants included in this umbrella review may differ from that detailed in the published review. M.G.T.O. extracted data for all studies, with 20% independently extracted by S.B. Any disagreements were resolved by consensus.

### Quality assessment

The methodological quality of each included review was assessed using the critical appraisal tool AMSTAR 2.^30^ Of the 16 included domains, 7 are regarded as critical (items 2, 4, 7, 9, 11, 13, and 15).^30^ Each item was rated “yes,” “partial yes,” or “no,” To assess overall confidence in the underlying review, a score of “no” on a critical domain was defined as a critical flaw; on a non-critical domain it was defined as a non-critical weakness. Overall confidence in findings of a particular systematic review was rated as critically low (multiple critical flaws with or without non-critical weaknesses), low (one critical flaw with or without non-critical weakness), moderate (multiple non-critical weakness), or high (no or one non-critical weakness).^30^

The quality of meta-analyses was further appraised. First, we assessed whether the included review assessed publication bias and, if so, whether there was evidence of small-sample bias or asymmetry in funnel plot distributions. Second, we evaluated heterogeneity using statistical approaches, subgroup analyses, or meta-regression techniques. Statistical heterogeneity was measured using the *I*² statistic. The *I*² measures percentage of total variability due to between-study heterogeneity, rather than chance, with the impact of between-study heterogeneity small if *I*² is close to 0% and large if *I*² is close to 100%, with *I*² > 50% interpreted as meaning that the effect size varies substantively across studies. However, *I*² is not an absolute measure of heterogeneity; hence, heterogeneity from.other sources, for example, design-related heterogeneity or study-level covariates, was examined through subgroup analyses or meta-regression techniques. In addition, to comprehensively examine the clinical implications of heterogeneity and determine how much the true effect sizes varied, prediction intervals were calculated (Appendix 1 in Supplementary Methods). A 95% prediction interval estimates where the true summary effect is expected to fall for 95% of comparable populations.^59,60^ A prediction interval is statistically significant when both the 95% CIs do not cross the null.^60^

Each identified meta-analysis was assigned a score on five criteria: publication bias (no = 1, yes = 0), statistical heterogeneity (<50% = 1, ≥50–75% = 0.5, >75% = 0), investigation of heterogeneity from other sources (subgroup/meta-regression techniques = 1, no = 0), prediction intervals (presented and rejects the null hypothesis = 1, presented but accepts the null hypothesis = 0.5, prediction interval not presented and when calculated accepts the null hypothesis = 0), and AMSTAR 2 rating (high = 1, moderate = 0.5, low = 0, critically low = 0). The five quality analysis scores were summed to determine an aggregate quality score within the range of 0–5. Composite scores of 4 or 5 indicate high quality of evidence for the respective review, with scores of 3 indicating moderate confidence, and scores of 2 or less indicating low confidence. Systematic reviews were assessed using only AMSTAR 2 ratings. The risk-of-bias plot was created using the robvis-package.^61^

### Data analysis

Where presented, all effect sizes and confidence intervals (except hazard ratios, which cannot be transformed) were converted to risk ratios for better communication of research findings^62^ and to enable comparison across outcomes (Appendix 2 in Supplementary Methods; packages ‘DescTools’ and ‘effectsize’ were used). Risk estimates of health outcomes following TBI were visually displayed on a forest plot using the ‘metafor’ package^63^ in R Studio.^64^

We calculated PAFs for each health outcome using Levin’s formula^65^ as most included reviews reported unadjusted effect sizes and presented only summary data. The PAF illustrates the percentage of all cases of the adverse outcome in the population attributable to a specific exposure, in this case TBI.^66^ We calculated the PAF using a conservative 12% prevalence estimate of TBI.^67,68^ It assumes causality and can be interpreted as the maximum possible impact of preventing all TBIs for an outcome.

Finally, a narrative synthesis of findings from all eligible papers was conducted. The evidence for an association between TBI and various health outcomes was assessed across severity and vulnerable subpopulations. Vulnerable subpopulations include older adults for whom increased fragility increases the risk of injury and post-recovery functional decline;^2,19^ children and adolescents where injury to the developing brain can disrupt development;^2^ athletes who are vulnerable to repetitive head impacts in sport;^2^ survivors of intimate partner violence who are subjected to environmental factors that can increase the risk of assault;^19^ and military personnel and veterans who can be vulnerable to complex injury mechanisms, including concussive blast TBI and comorbid extracranial polytrauma.^2^

### Reporting summary

Further information on research design is available in the Nature Portfolio Reporting Summary linked to this article.

## Supplementary information

Supplementary Information Supplementary Tables 1–8 and Appendices 1 and 2.

Reporting Summary

## Data Availability

All data were attained from published systematic reviews and meta-analyses and are cited in the text and in the reference list. The study protocol was registered with PROSPERO under registration number CRD42023432255.
