# Supplementary material for: An umbrella review of health outcomes following traumatic brain injury
Source: Nat Ment Health. 2025 Jan 3;3(1):83–91. doi: 10.1038/s44220-024-00356-5 (PMC11717692; doi:10.1038/s44220-024-00356-5)
Supplement: Supplementary file 1 — Supplementary Tables 1–8 and Appendices 1 and 2. [file 44220_2024_356_MOESM1_ESM.pdf]

nature mental health

Supplementary information https://doi.org/10.1038/s44220-024-00356-5

An umbrella review of health outcomes following traumatic brain injury

In the format provided by the authors and unedited

Supplementary Information

An Umbrella Review of Diagnosed Health Outcomes Following Traumatic Brain Injury

Supplementary Methods

Appendix 1. Statistical methods for quality assessment………….……………………………………. Page 2

Appendix 2. Statistical methods for data analysis………….. Page 3–4

Supplementary Tables

Table S1. Prisma research reporting guideline checklist ......…………………………………………. Page 5–8

Table S2. Search strategy and terms... Page 9–10

Table S3. Study characteristics table... .... Page 11–16

Table S4. Effect of mild traumatic brain injury on health outcomes and population attributable fractions.. Page 17

Table S5. Effect of moderate-severe traumatic brain injury on health outcomes and population attributable fractions... Page 17

Table S6. AMSTAR 2 ratings for each review.. Page 18–19

Table S7: Quality analyses for meta-analyses of health outcomes following traumatic brain injury...... Page 20

Table S8. Summary of evidence.. Page 21

Supplementary References…………………………………………………………………………….. **Page 22–25**

Appendix 1. Statistical methods for quality assessment

AMSTAR 2. All included studies were critically appraised using AMSTAR 2. If a meta-analysis was not performed, the items covering the appropriateness of the meta-analytical methods (items 11, 12, and 15) were deemed not applicable for systematic reviews and thus excluded when assessing overall confidence. One adaption to the standard AMSTAR 2 criteria was made. Item 7, which refers to the inclusion of a complete list of potentially relevant studies with justification for the exclusion of each, was not practical in most cases. Instead, item 7 was amended to state that a summary of the reports excluded (with reasons), in the form of PRISMA flow diagram or equivalent description in the results would suffice.

Statistical heterogeneity. When heterogeneity was reported by a meta-analysis as Q it was converted to I^2 with the following formula.[1]

$$I^2 = \left(\frac{Q - df}{Q}\right) * 100$$

Prediction Intervals. When sufficient information was available, prediction intervals were calculated using the following formula.[2]

$$P1\ lower = exp\left(\ln(OR) - t_{1-\frac{0.05}{2}} * \sqrt{(\tau^2 + SE^2)}\right)$$

$$PI\ upper = exp\left(\ln(OR) + t_{1-\frac{0.05}{2}} * \sqrt{(\tau^2 + SE^2)}\right)$$

SE^2 is the standard error of the natural logarithm of OR and was calculated using the following formula:

$$SE^2 = \frac{\Delta \log limits\ of\ the\ CI}{3.92} = \frac{ln(OR\ upper) - ln(OR\ lower)}{3.92}$$

τ^2 is a direct estimate of the between-study variation, however, was often not reported in favour of I^2. The following formula was used to convert τ^2 into I^2.

$$\tau^2 = s^2 * \frac{I^2}{(100 - I^2)}$$

Where s^2 is the study variance, equal to $\frac{\sum w_i(k-1)}{(\sum w_i)^2 - \sum w_i{}^2}$, and w_i equals the inverse of the study variance and k equals the number of studies.[2]

Appendix 2. Statistical methods for data analysis.

Effect sizes and confidence intervals were converted to risk ratios (RRs) for data analysis to enable comparison across outcomes – excluding for hazard ratios (HR) which cannot be converted. 'ORToRelRisk' from the 'DescTools' Package[3] in R Studio[4] was used to convert odds ratios (OR), and their confidence intervals, to RR. 'ORToRelRisk' transforms the OR to RR using the following formula,[5] where p_0 refers to the baseline risk.

$$RR = \frac{OR}{(1 - p_0) + (p_0 * OR)}$$

The base-rate prevalence of epilepsy, multiple sclerosis, psychosis/schizophrenia, Parkinson's disease, depression, and attention deficit-hyperactivity disorder was extracted from the Global Burden of Disease Study (GBD) 2019.[6] Base-rate prevalence of post-traumatic stress disorder was calculated using estimations from World Mental Health Surveys.[7] A crude worldwide prevalence rate of amyotrophic lateral sclerosis was extracted from a meta-analysis of 58 studies.[8] An estimation of violent crime rate was extracted using data from Office of National Statistics (ONS).[9]

Prevalence ratios (PR) were converted in RR using the following formula, based on the assumption that the prevalence in the control group is a good approximation of the population prevalence:

$$RR = 1 - p_r + (p_r * PR)$$

Where p_r refers to the prevalence in reference group.

Measures of standardised difference for continuous outcomes were considered approximately equivalent in large samples (where the degrees of freedom > 10).[10] We converted Cohen's d into OR using the 'effectsize' package,[11] before being further converting the effect size into RR using the procedure stated above.

The 'effectsize' package converts Cohen's d to OR using the following formula:[12]

$$OR = exp(d * \frac{\pi}{\sqrt{3}})$$

Variance of Cohen's d was equal to $V_{OR} = \exp(V_d \frac{\pi}{3})$. Estimations for the prevalence of olfactory dysfunction were extracted from a recent meta-analysis.[13]

Although one meta-analysis[14] provided effects sizes for postural control, these effect sizes could not be converted into risk ratios and presented on the risk estimates figure. However, the effect sizes extracted from the articles are presented in the study characteristics table (Table S2).

The population attributable fractions and confidence intervals for each health outcome were calculated using the following formulas,[15] where *p* refers to the prevalence in the population, and *RR* is the risk ratio.

$$PAF = \frac{p * (RR - 1)}{p * (RR - 1) + 1}$$

$$CI\ lower = \frac{p * (RR - 1\,) - z * \sqrt{\frac{p * (1 - p) * (RR - 1)^2}{n}}}{p * (RR - 1) + 1}$$

$$CI\ upper = \frac{p * (RR - 1\,) + z * \sqrt{\frac{p * (1 - p) * (RR - 1)^2}{n}}}{p * (RR - 1) + 1}$$

We calculated population attributable fractions using the conservative 12% prevalence estimate.[16] Prior research has indicated that the majority of individuals who have sustained TBI suffer from mild TBIs.[17,18] However, accurate data for TBI prevalence is limited,[19] and little meta-analytic evidence has sought to stratify by severity. Therefore, we have chosen to use the widely cited prevalence estimate of 12% for all PAF calculations. We note that the PAF for moderate-severe populations are likely to be overestimated, and the PAF for mild TBI may be underestimated, however this remained the most robust approach for calculating PAFs given the limited data. Further, Levin's approach presents some strong assumptions, which if violated may lead to biased estimates of the PAF, however due to the limited information available from the included systematic reviews and meta-analyses, this remained the most accessible approach for our analysis.

Table S1. PRSIMA Research Reporting Guideline checklist

PRISMA 2020 Main Checklist

Topic	No.	Item	Location where item is reported
**TITLE**			
**Title**	1	Identify the report as a systematic review.	Title
**ABSTRACT**			
**Abstract**	2	See the PRISMA 2020 for Abstracts checklist	
**INTRODUCTION**			
**Rationale**	3	Describe the rationale for the review in the context of existing knowledge.	Intro para 1
**Objectives**	4	Provide an explicit statement of the objective(s) or question(s) the review addresses.	Intro para 1 & 2
**METHODS**			
**Eligibility criteria**	5	Specify the inclusion and exclusion criteria for the review and how studies were grouped for the syntheses.	Methods para 3, 4 & 5
**Information sources**	6	Specify all databases, registers, websites, organisations, reference lists and other sources searched or consulted to identify studies. Specify the date when each source was last searched or consulted.	Methods para 2
**Search strategy**	7	Present the full search strategies for all databases, registers and websites, including any filters and limits used.	Supplement Material Table S2
**Selection process**	8	Specify the methods used to decide whether a study met the inclusion criteria of the review, including how many reviewers screened each record and each report retrieved, whether they worked independently, and if applicable, details of automation tools used in the process.	Methods para 5 & 6
**Data collection process**	9	Specify the methods used to collect data from reports, including how many reviewers collected data from each report, whether they worked independently, any processes for obtaining or confirming data from study investigators, and if applicable, details of automation tools used in the process.	Methods para 7
**Data items**	10a	List and define all outcomes for which data were sought. Specify whether all results that were compatible with each outcome domain in each study were sought (e.g. for all measures, time points, analyses), and if not, the methods used to decide which results to collect.	Methods para 3, 4
	10b	List and define all other variables for which data were sought (e.g. participant and intervention characteristics, funding sources). Describe any assumptions made about any missing or unclear information.	Methods para 7
**Study risk of bias assessment**	11	Specify the methods used to assess risk of bias in the included studies, including details of the tool(s) used, how many reviewers assessed each study and whether they worked independently, and if applicable, details of automation tools used in the process.	Methods para 8
**Effect measures**	12	Specify for each outcome the effect measure(s) (e.g. risk ratio, mean difference) used in the synthesis or presentation of results.	Methods para 11, Supplementary Materials Appendix 2
**Synthesis methods**	13a	Describe the processes used to decide which studies were eligible for each synthesis (e.g. tabulating the study intervention characteristics and comparing against the planned groups for each synthesis (item 5)).	Methods para 11 & 13

Topic	No.	Item	Location where item is reported
	13b	Describe any methods required to prepare the data for presentation or synthesis, such as handling of missing summary statistics, or data conversions.	Methods para 11 Supplementary Materials Appendix 2
	13c	Describe any methods used to tabulate or visually display results of individual studies and syntheses.	Methods para 11
	13d	Describe any methods used to synthesize results and provide a rationale for the choice(s). If meta-analysis was performed, describe the model(s), method(s) to identify the presence and extent of statistical heterogeneity, and software package(s) used.	Methods para 11
	13e	Describe any methods used to explore possible causes of heterogeneity among study results (e.g. subgroup analysis, meta-regression).	Methods para 9, 10 & 13
	13f	Describe any sensitivity analyses conducted to assess robustness of the synthesized results.	Methods 10 | Supplementary Appendix 2
**Reporting bias assessment**	14	Describe any methods used to assess risk of bias due to missing results in a synthesis (arising from reporting biases).	NA
**Certainty assessment**	15	Describe any methods used to assess certainty (or confidence) in the body of evidence for an outcome.	Methods para 10
**RESULTS**			
**Study selection**	16a	Describe the results of the search and selection process, from the number of records identified in the search to the number of studies included in the review, ideally using a flow diagram.	Results para 1
	16b	Cite studies that might appear to meet the inclusion criteria, but which were excluded, and explain why they were excluded.	Figure 1
**Study characteristics**	17	Cite each included study and present its characteristics.	Supplementary Materials Table S2
**Risk of bias in studies**	18	Present assessments of risk of bias for each included study.	Supplementary Materials Table S6 & S7
**Results of individual studies**	19	For all outcomes, present, for each study: (a) summary statistics for each group (where appropriate) and (b) an effect estimate and its precision (e.g. confidence/credible interval), ideally using structured tables or plots.	Figure 2
**Results of syntheses**	20a	For each synthesis, briefly summarise the characteristics and risk of bias among contributing studies.	Results para 7 & 8
	20b	Present results of all statistical syntheses conducted. If meta-analysis was done, present for each the summary estimate and its precision (e.g. confidence/credible interval) and measures of statistical heterogeneity. If comparing groups, describe the direction of the effect.	Results para 3, 4 5
	20c	Present results of all investigations of possible causes of heterogeneity among study results.	Results para 7 & 8
	20d	Present results of all sensitivity analyses conducted to assess the robustness of the synthesized results.	Results para 4
**Reporting biases**	21	Present assessments of risk of bias due to missing results (arising from reporting biases) for each synthesis assessed.	NA
**Certainty of evidence**	22	Present assessments of certainty (or confidence) in the body of evidence for each outcome assessed.	Results para 7
**DISCUSSION**			

Topic	No.	Item	Location where item is reported
**Discussion**	23a	Provide a general interpretation of the results in the context of other evidence.	Discussion para 1
	23b	Discuss any limitations of the evidence included in the review.	Discussion para 2, 3, 4, 5, 6 & 7
	23c	Discuss any limitations of the review processes used.	Discussion para 10
	23d	Discuss implications of the results for practice, policy, and future research.	Conclusion para 1 & 2
**OTHER INFORMATION**			
**Registration and protocol**	24a	Provide registration information for the review, including register name and registration number, or state that the review was not registered.	Methods para 1
	24b	Indicate where the review protocol can be accessed, or state that a protocol was not prepared.	Methods para 1
	24c	Describe and explain any amendments to information provided at registration or in the protocol.	NA
**Support**	25	Describe sources of financial or non-financial support for the review, and the role of the funders or sponsors in the review.	Funding & Acknowledgements
**Competing interests**	26	Declare any competing interests of review authors.	Declarations of interest
**Availability of data, code and other materials**	27	Report which of the following are publicly available and where they can be found: template data collection forms; data extracted from included studies; data used for all analyses; analytic code; any other materials used in the review.	Data sharing

PRIMSA Abstract Checklist

Topic	No.	Item	Reported?
**TITLE**			
**Title**	1	Identify the report as a systematic review.	Yes
**BACKGROUND**			
**Objectives**	2	Provide an explicit statement of the main objective(s) or question(s) the review addresses.	Yes
**METHODS**			
**Eligibility criteria**	3	Specify the inclusion and exclusion criteria for the review.	Yes
**Information sources**	4	Specify the information sources (e.g. databases, registers) used to identify studies and the date when each was last searched.	Yes
**Risk of bias**	5	Specify the methods used to assess risk of bias in the included studies.	Yes
**Synthesis of results**	6	Specify the methods used to present and synthesize results.	Yes
**RESULTS**			
**Included studies**	7	Give the total number of included studies and participants and summarise relevant characteristics of studies.	Yes

Topic	No.	Item	Reported?
**Synthesis of results**	8	Present results for main outcomes, preferably indicating the number of included studies and participants for each. If meta-analysis was done, report the summary estimate and confidence/credible interval. If comparing groups, indicate the direction of the effect (i.e. which group is favoured).	Yes
**DISCUSSION**			
**Limitations of evidence**	9	Provide a brief summary of the limitations of the evidence included in the review (e.g. study risk of bias, inconsistency and imprecision).	Yes
**Interpretation**	10	Provide a general interpretation of the results and important implications.	Yes
**OTHER**			
**Funding**	11	Specify the primary source of funding for the review.	Yes
**Registration**	12	Provide the register name and registration number.	Yes

From: Page MJ, McKenzie JE, Bossuyt PM, Boutron I, Hoffmann TC, Mulrow CD, et al. The PRISMA 2020 statement: an updated guideline for reporting systematic reviews. MetaArXiv. 2020, September 14. DOI: 10.31222/osf.io/v7gm2. For more information, visit: www.prisma-statement.org

Table S2. Search strategy and terms.

**Embase 1974 to present**		
#	**Query**	**Results from 17 May 2023**
1	exp traumatic brain injury/	67,279
2	(TBI or "Brain Injur*" or "Diffuse Axonal Injur*" or "diffuse cerebral" or DAI or "Head Injur*" or "Head Trauma" or (cerebral adj (trauma* or injur*)) or (Traumatic adj (Brain or Cerebr*)) or ((Posttraumatic or Traumatic) adj Encephalopath*) or Concuss*).ti,ab.	186,382
3	1 or 2	197,951
4	exp meta analysis/	293,559
5	((systematic adj2 review) or meta analy* or metaanaly* or (methodologic* adj (review or overview)) or (quantitative adj (review or overview))).ti,ab,kw.	541,947
6	4 or 5	585,144
7	3 and 6	4,593
8	(protocol or treat* or intervention*).ti.	2,292,344
9	7 not 8	3,981

**PsycINFO 1806 to present**		
#	**Query**	**Results from 17 May 2023**
1	exp traumatic brain injury/	22,993
2	(TBI or "Brain Injur*" or "Diffuse Axonal Injur*" or "diffuse cerebral" or DAI or "Head Injur*" or "Head Trauma" or (cerebral adj (trauma* or injur*)) or (Traumatic adj (Brain or Cerebr*)) or ((Posttraumatic or Traumatic) adj Encephalopath*) or Concuss*).ti,ab.	40,705
3	1 or 2	41,628
4	meta analysis/ or "systematic review"/	6,006
5	((systematic adj2 review) or meta analy* or metaanaly* or (methodologic* adj (review or overview)) or (quantitative adj (review or overview))).ti,ab.	81,439
6	4 or 5	81,998
7	3 and 6	1,069
8	(protocol or treat* or intervention*).ti.	260,103
9	7 not 8	900

**Global Health <1973 to 2023 Week 19>**		
#	**Query**	**Results from 17 May 2023**
1	(TBI or "Brain Injur*" or "Diffuse Axonal Injur*" or "diffuse cerebral" or DAI or "Head Injur*" or "Head Trauma" or (cerebral adj (trauma* or injur*)) or (Traumatic adj (Brain or Cerebr*)) or ((Posttraumatic or Traumatic) adj Encephalopath*) or Concuss*).ti,ab.	7,726
2	meta analysis/ or "systematic review"/	74,443
3	((systematic adj2 review) or meta analy* or metaanaly* or (methodologic* adj (review or overview)) or (quantitative adj (review or overview))).ti,ab.	79,942
4	2 or 3	86,772
5	1 and 4	220
6	(protocol or treat* or intervention*).ti.	214,857
7	5 not 6	193

**Medline (Ovid MEDLINE® Epub Ahead of Print, In-Process & Other Non-Indexed Citations, Ovid MEDLINE® Daily and Ovid MEDLINE®) 1946 to present**		
#	**Query**	**Results from 17 May 2023**
1	exp brain injuries, diffuse/ or exp brain injuries, traumatic/	24,491
2	(TBI or "Brain Injur*" or "Diffuse Axonal Injur*" or "diffuse cerebral" or DAI or "Head Injur*" or "Head Trauma" or (cerebral adj (trauma* or injur*)) or (Traumatic adj (Brain or Cerebr*)) or ((Posttraumatic or Traumatic) adj Encephalopath*) or Concuss*).ti,ab.	131,029
3	1 or 2	133,391
4	meta-analysis/ or "systematic review"/	310,830

5	((systematic adj2 review) or meta analy* or metaanaly* or (methodologic* adj (review or overview)) or (quantitative adj (review or overview))).ti,ab,kw.	415,081
6	4 or 5	445,812
7	3 and 6	3,234
8	(protocol or treat* or intervention*).ti.	1,835,848
9	7 not 8	2,786

**Cochrane Database of Systematic Reviews**		
**#**	**Query**	**Results from 17 May 2023**
1	MeSH descriptor: [Brain Injuries, Traumatic] explode all trees	1,345
2	(TBI or "Brain Injur*" or "Diffuse Axonal Injur*" or "diffuse cerebral" or DAI or "Head Injur*" or "Head Trauma" or (cerebral adj (trauma* or injur*)) or (Traumatic adj (Brain or Cerebr*)) or ((Posttraumatic or Traumatic) adj Encephalopath*) or Concuss*):ti,ab,kw	15,664
3	1 or 2	15665
4	(treat* or intervention*):ti	374,601
	3 not 4	13212
	Cochrane Reviews	168

**Google Scholar**		
**#**	**Query**	**Results 02 Nov 2023**
1	(Traumatic Brain Injury or TBI)	-
2	(Meta-analysis or systematic review)	-
3	Outcome	-
	1 and 2 and 3	First 100 results screened

Table S3. Study characteristics table

Author(s) and date	Study design	Review (year range)	TBI definition and severity	Population	Number of primary studies	Sample size	Type of controls	Outcome	Number of databases searched	Country	Quality assessment	Summary statistic	Summary effect size (95% CI)	I^2
Asarnow et al. (2021)[20]	Meta-analysis	1981-2020	TBI as stratified by concussion, mild, moderate and severe	Paediatric	24	12374 TBI; 43491 controls	Non-injured controls and other injured controls	ADHD	3	NR	Newcastle Ottowa Scale	Odds Ratio	Mild = 1·18 (0·32-3·12)<br><br>Moderate = 3·78 (0·93-10·33)<br><br>Severe = 6·70 (2·02-16·82)	NR
Balabandian et al. (2023)[21]*	Meta-analysis	1991-2021	Any head injury leading to LOC	Elderly	23	145168 TBI; 377386 controls	Healthy controls (general population)	Parkinson’s Disease	3	North America, Europe, Brazil, Taiwan	Newcastle Ottowa Scale	Odds Ratio	Any TBI = 1·50 (1·12-1·83)	95·8%
Broglio et al. (2008)[14]	Meta-analysis	1996-2003	Any concussion or mild TBI	Sports	6	338	NR	Postural Control	2	NR	A 15-item scale[22]	Aggregated mean effect size	Initial assessment = -2·56 (-6·44, 1·32)<br><br>After 14 days = -1·16 (-2·59, 0·28)	Initial assessment = 99·8%<br>After 14 days = 97·8%

Cancelliere et al. (2023)[23]	Meta-analysis	2010-2021	Mild TBI as defined by the ACRM or similar criteria	General	43 (7 meta-analysis)	11742 TBI; 3004 controls	Orthopaedic-injured controls	Post-Concussion Symptoms	5	USA, France, Norway, Australia	Scottish Intercollegiate Guidelines Network	Prevalence Ratio	Mild = 1·57 (1·22-2·02)	80%
Dever et al. (2022)[24]	Systematic Review	2003-2021	Mild, moderate, or severe injury to the head that results in acute or chronic symptoms	General	13	314 TBI; 233 controls	Health controls	Gait Impairment	4	NR	NR			
Emery et al. (2016)[25]	Systematic Review	1997-2012	Mild TBI	Paediatric	8	1664 TBI; 1343 controls	Healthy controls, non-injured/orthopaedic controls	Depression, Anxiety, ODD, PTSD, Autism, Substance Abuse	9	USA, UK, New Zealand	Down and Black Checklist			
Esterov et al. (2023)[26]	Meta-analysis	2011-2020	TBI of any severity as decided by ICD-9 criteria	General	8	619992 TBI; 1692865 controls	Mixed (pre-post measurements, non-TBI and other injured controls)	Stroke	5	Taiwan, USA	Newcastle Ottowa Scale	Hazard Ratio	Any stroke = 2·06 (1·28-3·32)<br><br>Ischemic stroke = 1·38 (1·24-1·54)	100%
Fazel et al. (2009)[27]	Meta-analysis	1992-2006	Any head injury	General, and forensic	6	2420 TBI; 717 controls	Non affected controls	Violence	4	Australia, Finland, USA	Sackett quality assessment	Odds Ratio	Total sample = 1·66 (1·12-2·31)<br><br>Male only = 1·46	0%

													(1·00-2·13) Non-prison samples = 1·64 (1·07-2·52)	
Fralick et al. (2018)[28]	Meta-analysis	2001-2016	Concussion (mild TBI) defined by a transient disturbance of neurological function caused by acute trauma	General, Military	6	712235 TBI; 13651021 controls	Non-affected controls	Suicide	4	Denmark, USA, Sweden, Canada	Newcastle Ottowa Scale	Risk Ratio	RR = 2·03 (1·47-2·80) adjusted RR = 2·10 (1·40-3·13)	96% (adjusted = 94%)
Gardner et al. (2023)[29]	Meta-analysis	1990-2019	Any head injury/ trauma and TBI	General and military	32	7634844	NR	Dementia	3	North America, Europe, Australia, Asia	Newcastle Ottowa Scale	Risk Ratio	1·66 (1·42-1·93)	98·7%
Grants et al. (2017)[30]	Systematic Review	2005-2015	Concussion as defined using American Academy of Neurology Practice Parameter or 3rd International Consensus	Paediatric and sport	10	204 TBI; 204 controls	Non-concussed controls (matched on age, sex, mass height)	Gait Impairment (under dual-task conditions)	6	NR	Joanna Briggs Institute Critical Appraisal Checklist			

Iljazi et al. (2020)[31]	Systematic Review	1998-2013	Exposure to blunt trauma, penetrating injury or blast injury to the head, of any severity	Paediatric	2	132 TBI; 80 controls	Orthopaedic-injured controls	PTSD	2	Australia, USA	Newcastle Ottowa Scale			
Liu et al. (2021)[32]	Meta-analysis	1980-2020	Any head injury defined on medical records, military records, questionnaire of self-report	General	14	10703 TBI; 2159324	Hospital-based and population-based controls	Amyotrophic Lateral Sclerosis	1	Europe, USA, China	Newcastle Ottowa Scale	Odds Ratio	Any = 1·38 (1·20-1·60)<br><br>Severe = 1·69 (1·27-2·23)	11%
McElvenny et al. (2021)[33]	Meta-analysis	1970-2015	Any head injury involving LOC, amnesia, skull fracture, or requiring medical treatment	General	13	626707	Hospital-based and population-based controls	Brain Cancer	9	NR	Newcastle Ottowa Scale	Risk Ratio	1·40 (1·11-1·77)	76%
O'Neil et al. (2014)[34]	Systematic Review	2008-2019	Mild TBI as defined by the VA and Department of Defence common definition	Military	11	186881 TBI; 544333 controls	Non-mTBI injured and non-injured	Chronic Pain	8	USA	Risk of Bias in Non-randomized Studies of Interventions, Newcastle Ottawa Scale			
Perry et al. (2016)[35]	Meta-analysis	2001-2010	TBI without structural lesions,	General	10	2996 TBI; 11656 controls	NR	Depression	1	NR	NR	Odds Ratio	2·14 (1·65-2·77)	NR

			specifically mild TBI											
Ramirez et al. (2022)[36]	Meta-analysis	2016-2019	Sports-related concussion defined as traumatically induced alteration of mental status	Sports	7 (5 meta-analysis)	366 TBI; 513 controls	Collegiate athletes without concussions	Musculoskeletal Injury	3	NR	Downs and Black Checklist	Risk Ratio	1·58 (1·20-1·93)<br>First 90 days RTP = 2·20 (1·58-3·05)<br>One year RTP = 1·26 (0·98-1·61)	12·74%
Rutherford et al. (2009)[37]	Systematic Review	1971-2005	TBI due to an external force	General and military	12	NR	Mixed (general-population, non-injured, peripheral nerve injured)	Mortality	NR	Australia, USA, Germany	NR			
Sabol et al. (2021)[38]	Systematic Review	2009-2018	Sports-related concussion (mild TBI) due to acceleration of deceleration of the head	Sports	6	192 TBI; 632 controls	Musculoskeletal injury	Depression & Anxiety	5	NR	Joanna Brigs Institute Crititcal Appraisal Tool			
Sui et al. (2022)[39]	Meta-analysis	2009-2021	Any TBI	General	10	2508948 TBI; 7352205 controls	Mixed (orthopaedic controls, non-TBI controls, general)	Epilepsy	3	USA, Sweden, Denmark, Finland, Israel, China	Newcastle Ottowa Scale	Odds Ratio	4·25 (1·77-10·25)	100%

Tai et al. (2022)[40]	Meta-analysis	1985-2020	Moderate to severe TBI based on the 2021 Department of Veterans Affairs / Department of Defence Clinical Practice Guideline criteria for TBI	General	11	429 TBI; 559 controls	Healthy controls	Olfactory Dysfunction	5	NR	Newcastle Ottowa Scale	Hedge's g	-2·43 (-3·16, -1·69)	94·64%
Van Praag et al. (2019)[41]	Meta-analysis	1999-2014	Any TBI	General	14	2132 TBI; 4244 controls	Non-TBI trauma controls, healthy controls	PTSD	6	Australia, USA, France	Methodological evaluation of Observational REsearch	Odds Ratio	Any TBI = 1·73 (1·21-2·47)<br><br>mTBI = 1·56 (1·06-2·30)	64% (mTBI = 72%)
Warren et al. (2013)[42]	Meta-analysis	1952-2009	Any cranial/head injury or traumatic brain injury	General	5	NR	NR	Multiple Sclerosis	15	USA, UK, Canada, Denmark	Newcastle Ottowa Scale	Odds Ratio	1·46 (0·83-2·55)	25%
Yau et al. (2023)[43]	Meta-analysis	1987-2022	Any TBI which occurred before the age of 18	Paediatric	10 (8 meta-analysis)	153757 TBI; 325929 controls	NR	Psychosis and Psychotic Symptoms	2	USA, Canada, Denmark, Finland, Norway, Sweden	Kmet et al· (2014) Quality Assessment Scale	Odds Ratio	1·80 (1·11-2·95)<br><br>Excluding low quality = 1·43 (1·04-1·98)	69% (excluding low quality = 64%)

NR = not reported· LOC = loss of consciousness· ACRM = American Congress of Rehabilitation Medicine· For some articles, not all primary studies met inclusion criteria, therefore where possible data was extracted for the studies/ subgroups which met inclusion criteria· *Inconsistent reporting.

Table S4. Effect of mild traumatic brain injury on health outcomes and population attributable fractions

Health outcome	Population	Risk ratio	95% CI	95% Prediction interval	Population attributable fraction (%)	95% CI
Depression	General	2·06	1·61—2·62	··	11·3	10·79—11·78
Suicide	General	2·03	1·47—2·80	0·77—5·38	11·0	11·98—11·02
Musculoskeletal Injury	General	1·58	1·30—1·93	··	6·5	2·70—10·32
PTSD	General	1·53	1·06—2·19	0·50—4·90	6·0	4·88—7·08
Post-Concussion Symptoms	General	1·40	1·29—1·50	1·14—1·71	4·6	3·27—5·89
ADHD	Paediatric	1·18	0·32—3·05	··	2·1	-3·41—7·64

Table S5. Effect of moderate-severe traumatic brain injury on health outcomes and population attributable fractions

Health outcome	TBI severity	Population	Risk ratio	95% CI	95% Prediction interval	Population attributable fraction (%)	95% CI
ADHD	Severe	Paediatric	6·29	2·00—14·30	··	38·8	38·67—38·99
Olfactory dysfunction	Moderate-Severe	General	4·32	3·29—4·34	4·21—4·43	28·9	27·88—29·10
ADHD	Moderate	Paediatric	3·66	0·93—9·34	··	24·2	21·98—26·41
Amyotrophic Lateral Sclerosis	Severe	General	1·69	1·27—2·23	1·07—2·67	7·6	7·11—8·19

Table S6. AMSTAR 2 ratings for each review

Author(s) and date	Item 1	Item 2	Item 3	Item 4	Item 5	Item 6	Item 7	Item 8	Item 9	Item 10	Item 11	Item 12	Item 13	Item 14	Item 15	Item 16	AMSTAR 2 rating
Asarnow et al· (2021)	N	N	Y	N	Y	N	Y	N	Y	N	Y	N	N	Y	N	Y	critically low
Balabandian et al· (2023)	Y	N	N	N	Y	Y	N	N	Y	N	Y	N	N	N	N	Y	critically low
Broglio et al· (2008)	Y	N	N	N	N	N	Y	N	N	N	N	N	N	N	Y	Y	critically low
Cancelliere et al· (2023)	N	Y	Y	PY	Y	Y	Y	N	Y	N	Y	N	Y	Y	N	Y	low
Dever et al· (2022)	N	N	N	N	Y	N	Y	Y	N	N	NA	NA	N	Y	NA	Y	critically low
Emery et al· (2016)	N	Y	N	N	Y	Y	N	N	Y	N	NA	NA	Y	Y	NA	Y	critically low
Esterov et al· (2023)	Y	Y	N	PY	Y	N	Y	Y	Y	N	Y	N	N	Y	N	Y	critically low
Fazel et al· (2009)	Y	N	N	PY	Y	N	N	Y	N	N	Y	N	Y	Y	Y	Y	critically low
Fralick et al· (2018)	Y	Y	N	N	Y	Y	Y	Y	Y	N	Y	Y	N	Y	Y	Y	critically low
Gardner et al· (2023)	Y	Y	N	PY	Y	N	Y	N	Y	N	Y	N	Y	Y	Y	Y	moderate
Grants et al· (2017)	Y	Y	Y	PY	Y	Y	N	Y	Y	N	NA	NA	N	N	NA	N	critically low
Iljazi et al· (2020)	Y	N	N	N	Y	Y	N	Y	Y	N	NA	NA	Y	Y	NA	Y	critically low
Liu et al· (2021)	N	N	N	N	Y	Y	Y	Y	Y	N	Y	N	Y	Y	Y	Y	critically low
McElvenny et al· (2021)	N	N	N	PY	Y	Y	PY	Y	Y	N	Y	N	N	Y	Y	Y	critically low

O'Neil et al· (2014)	Y	N	N	Y	Y	Y	Y	Y	Y	N	NA	NA	Y	Y	NA	Y	low
Perry et al· (2016)	Y	N	N	N	Y	N	N	N	N	N	Y	N	N	Y	Y	Y	critically low
Ramirez et al· (2022)	N	N	N	N	Y	N	Y	Y	Y	N	Y	N	N	N	N	Y	critically low
Rutherford et al· (2009)	N	N	N	N	N	N	N	PY	N	N	NA	NA	N	Y	NA	N	critically low
Sabol et al· (2021)	Y	Y	N	PY	Y	N	Y	Y	Y	N	NA	NA	N	Y	NA	Y	low
Sui et al· (2022)	Y	N	N	N	Y	N	Y	N	Y	N	Y	N	N	Y	Y	N	critically low
Tai et al· (2022)	Y	N	N	N	Y	N	Y	N	Y	N	Y	N	N	Y	Y	N	critically low
Van Praag et al· (2019)	N	Y	N	PY	Y	N	N	N	Y	N	N	Y	Y	Y	N	Y	critically low
Warren et al· (2013)	Y	N	Y	Y	Y	Y	Y	N	Y	N	Y	Y	Y	Y	N	Y	critically low
Yau et al· (2023)	Y	Y	N	Y	Y	Y	Y	N	Y	N	Y	Y	Y	Y	Y	Y	moderate

Note· Y = yes, N = no, PY = partial yes· NA = not applicable· See https://amstar·ca/docs/AMSTAR%202-Guidance-document·pdf for a list of the AMSTAR questions· If a meta-analysis was not performed, the items covering the appropriateness of the meta-analytical methods (items 11, 12, and 15) were not applicable and thus excluded when assessing overall confidence· Item 7 was amended to state that a summary of the reports excluded (with reasons), in the form of PRISMA flow diagram or equivalent description in the results would suffice·

Table S7. Quality analyses for meta-analyses of health outcomes following traumatic brain injury.

**Author(s) and date**	**Outcome**	**Publication bias**	**Statistical heterogeneity**	**Assessed other sources of heterogeneity**	**Prediction interval excludes null value**	**AMSTAR**	**Overall score**	**Overall judgement**
Asarnow et al· (2021)	ADHD	··	··	No	··	0	0	Low
Balabandian et al· (2023)	Parkinson's Disease	Yes	Very high	Yes	No	0	1	Low
Broglio et al· (2008)	Postural Control	No	Very high	No	··	0	1	Low
Cancelliere et al· (2023)	Post-Concussion Symptoms	··	Very high	Yes	Yes	0	2	Low
Esterov et al· (2023)	Stroke	··	Very high	Yes	No	0	1	Low
Fazel et al· (2009)	Violence	Yes	Low	Yes	Yes	0	3	Moderate
Fralick et al· (2018)	Suicide	No	Very high	Yes	No	0	2	Low
Gardner et al· (2023)	Dementia	No	Very high	Yes	No	0·5	3	Moderate
Liu et al· (2021)	Amyotrophic Lateral Sclerosis	No	Low	Yes	Yes	0	4	High
McElvenny et al· (2021)	Brain Cancer	No	Very high	Yes	No	0	2	Low
Perry et al· (2016)	Depression	No	Very high	Yes	··	0	2	Low
Ramirez et al· (2022)	Musculoskeletal Injury	··	High	Yes	··	0	1·5	Low
Sui et al· (2022)	Epilepsy	Yes	Very high	Yes	··	0	1	Low
Tai et al· (2022)	Olfactory Dysfunction	Yes	Very high	Yes	Yes	0	2	Low
Van Praag et al· (2019)	PSTD	··	High	Yes	No	0	1.5	Low
Warren et al· (2013)	Multiple Sclerosis	··	Low	Yes	··	0	2	Low
Yau et al· (2023)	Psychosis	Yes	High	Yes	No	0·5	2	Low

Table S8. Summary of evidence

**Health Outcome**	**Author**	**Evidence of increased risk?**
Olfactory dysfunction	Tai et al. (2022)	PI = [4.21, 4.43]
Post-concussion symptoms	Cancelliere et al. (2023)	PI = [1.14, 1.71]
Amyotrophic lateral sclerosis	Liu et al. (2021)	PI_{severe} = [1.07, 2.67]<br>PI_{any} = [0.93, 1.96]
Violence perpetration	Fazel et al. (2009)	PI = [1.03, 2.67]
Attention deficit hyperactivity disorder	Asarnow et al (2021)	
Epilepsy	Sui et al. (2022)	
Depression	Perry et al. (2016), Sabol et al. (2021)	
Stroke	Esterov et al. (2023)	PI = [0.53, 7.95]
Suicide	Fralick et al. (2018)	PI = [0.77, 5.38]
Psychosis	Yau et al. (2023)	PI = [0.71, 4.54]
Dementia	Gardner et al. (2023)	
Musculoskeletal injury	Ramirez et al. (2022)	
Parkinson's disease	Balbandian et al. (2023)	PI = [0.66, 3.40]
Posttraumatic stress disorder	Iljazi et al. (2020), Van Praag et al. (2019)	PI = [0.50, 4.90]
Multiple sclerosis	Warren et al. (2013)	
Brain cancer	McElvenny et al. (2021)	PI = [0.63, 3.09]
Postural control	Broglio et al. (2008)	
Gait impairment	Dever et al. (2022), Grants et al. (2017)	
Chronic pain	O'Neil et al. (2014)	
Mortality	Rutherford et al. (2009)	
Anxiety	Emery et al. (2016), Sabol et al. (2021)	
Autism	Emery et al. (2016)	
Substance abuse	Emery et al. (2016)	
Oppositional defiant (or conduct) disorder	Emery et al. (2016)	

Table 2. Summary of evidence.

Note. PI = prediction intervals

Green shows clear evidence of an increased risk of the outcome following TBI. Yellow indicates that there is evidence suggesting an increased risk however substantiated conclusions could not be drawn. Red indicates insufficient evidence.

Supplementary References

1. Borenstein, M. Avoiding common mistakes in meta-analysis: Understanding the distinct roles of Q, I-squared, tau-squared, and the prediction interval in reporting heterogeneity. *Res. Synth. Methods* **15**, 354–368 (2024).
2. IntHout, J., Ioannidis, J. P. A., Rovers, M. M. & Goeman, J. J. Plea for routinely presenting prediction intervals in meta-analysis. *BMJ Open* **6**, e010247 (2016).
3. Andri Signorell. DescTools: Tools for Descriptive Statistics. https://andrisignorell.github.io/DescTools/ (2023).
4. RStudio Team. RStudio: Integrated Development for R. (2020).
5. Zhang, J. & Yu, K. F. What's the Relative Risk?A Method of Correcting the Odds Ratio in Cohort Studies of Common Outcomes. *JAMA* **280**, 1690–1691 (1998).
6. GBD 2019. GBD cause and risk summaries. *The Lancet* https://www.thelancet.com/gbd/summaries (2019).
7. Koenen, K. C. *et al.* Posttraumatic stress disorder in the World Mental Health Surveys. *Psychol. Med.* **47**, 2260–2274 (2017).
8. Xu, L. *et al.* Global variation in prevalence and incidence of amyotrophic lateral sclerosis: a systematic review and meta-analysis. *J. Neurol.* **267**, 944–953 (2020).
9. Office for National Statistics. Crime in England and Wales: Police Force Area data tables. https://www.ons.gov.uk/peoplepopulationandcommunity/crimeandjustice/datasets/policeforceareadatatables.
10. Borenstein, M. Effect sizes for continous data. in *The Handbook of Research Synthesis and Meta-Analysis* 221–235 (Russell Sage Foundation, New York, 1994).
11. Ben-Shachar, M., Lüdecke, D. & Makowski, D. effectsize: Estimation of Effect Size Indices and Standardized Parameters. *Open J.* **5**, 2815 (2020).
12. Michael Borenstein, Larry V. Hedges, Julian P T Higgins, & Hannah R. Rothstein. Converting Among Effect Sizes. in 45–49 (John Wiley & Sons, Ltd, Chichester, UK, 2009). doi:10.1002/9780470743386.ch7.
13. Desiato, V. M. *et al.* The Prevalence of Olfactory Dysfunction in the General Population: A Systematic Review and Meta-analysis. *Am. J. Rhinol. Allergy* **35**, 195–205 (2021).
14. Broglio, S. P. & Puetz, T. W. The Effect of Sport Concussion on Neurocognitive Function, Self-Report Symptoms and Postural Control. *Sports Med.* **38**, 53–67 (2008).
15. Levin, M. The occurrence of lung cancer in man. *Acta Unio Int Contra Cancrum* **9**, 531–941 (1953).

16. Frost, R. B., Farrer, T. J., Primosch, M. & Hedges, D. W. Prevalence of traumatic brain injury in the general adult population: a meta-analysis. *Neuroepidemiology* **40**, 154–159 (2013).
17. Sariaslan, A., Sharp, D. J., D'Onofrio, B. M., Larsson, H. & Fazel, S. Long-term outcomes associated with traumatic brain injury in childhood and adolescence: a nationwide Swedish cohort study of a wide range of medical and social outcomes. *PLoS Med.* **13**, e1002103 (2016).
18. Maas, A. I. R. *et al.* Traumatic brain injury: progress and challenges in prevention, clinical care, and research. *Lancet Neurol.* **21**, 1004–1060 (2022).
19. Maas, A. I. R. *et al.* Traumatic brain injury: integrated approaches to improve prevention, clinical care, and research. *Lancet Neurol.* **16**, 987–1048 (2017).
20. Asarnow, R. F., Newman, N., Weiss, R. E. & Su, E. Association of Attention-Deficit/Hyperactivity Disorder Diagnoses With Pediatric Traumatic Brain Injury: A Meta-analysis. *JAMA Pediatr.* **175**, 1009–1016 (2021).
21. Balabandian, M., Noori, M., Lak, B., Karimizadeh, Z. & Nabizadeh, F. Traumatic brain injury and risk of Parkinson's disease: a meta-analysis. *Acta Neurol. Belg.* **123**, 1225–1239 (2023).
22. Detsky, A. S., Naylor, C. D., O'Rourke, K., McGeer, A. J. & L'Abbé, K. A. Incorporating variations in the quality of individual randomized trials into meta-analysis. *J. Clin. Epidemiol.* **45**, 255–265 (1992).
23. Cancelliere, C. *et al.* Post-Concussion Symptoms and Disability in Adults With Mild Traumatic Brain Injury: A Systematic Review and Meta-Analysis. *J. Neurotrauma* **40**, 1045–1059 (2023).
24. Dever, A. *et al.* Gait Impairment in Traumatic Brain Injury: A Systematic Review. *Sensors* **22**, 1480 (2022).
25. Emery, C. A. *et al.* A Systematic Review of Psychiatric, Psychological, and Behavioural Outcomes following Mild Traumatic Brain Injury in Children and Adolescents. *Can. J. Psychiatry* **61**, 259–269 (2016).
26. Esterov, D., Sperl, M. A., Hines, E. A., Kinzelman Vesely, E. A. & Brown, A. W. Association Between Traumatic Brain Injury and Increased Risk of Stroke: A Systematic Review and Meta-analysis. *J. Head Trauma Rehabil.* **38**, E44 (2023).
27. Fazel, S., Philipson, J., Gardiner, L., Merritt, R. & Grann, M. Neurological disorders and violence: a systematic review and meta-analysis with a focus on epilepsy and traumatic brain injury. *J. Neurol.* **256**, 1591–1602 (2009).

28. Fralick, M. *et al.* Association of Concussion With the Risk of Suicide: A Systematic Review and Meta-analysis. *JAMA Neurol.* **76**, 144–151 (2019).

29. Gardner, R. C. *et al.* Systematic Review, Meta-Analysis, and Population Attributable Risk of Dementia Associated with Traumatic Brain Injury in Civilians and Veterans. *J. Neurotrauma* **40**, 620–634 (2023).

30. Grants, L., Powell, B., Gessel, C., Hiser, F. & Hassen, A. Gait deficits under dual – task conditions in the concussed adolescent and young athlete population: A systematic review. *Int. J. Sports Phys. Ther.* **12**, 1011–1022 (2017).

31. Iljazi, A. *et al.* Post-Traumatic Stress Disorder After Traumatic Brain Injury—A Systematic Review and Meta-Analysis. *Neurol. Sci.* **41**, 2737–2746 (2020).

32. Liu, G. *et al.* Head Injury and Amyotrophic Lateral Sclerosis: A Meta-Analysis. *Neuroepidemiology* **55**, 11–19 (2021).

33. McElvenny, D. M. *et al.* Systematic review of the epidemiology of a single physical trauma and cancer. *Trauma* **23**, 175–187 (2021).

34. O'Neil, M. *et al. Chronic Pain in Veterans and Servicemembers with a History of Mild Traumatic Brain Injury: A Systematic Review*. (2021).

35. Perry, D. C. *et al.* Traumatic brain injury is associated with subsequent neurologic and psychiatric disease: a meta-analysis. *J. Neurosurg.* **124**, 511–526 (2016).

36. Ramirez, V., McCann, R., Schussler, E. & Martinez, J. The Effect of Concussion History on Lower Extremity Injury Risk in College Athletes: A Systematic Review and Meta-Analysis. *Int. J. Sports Phys. Ther.* **17**, 753–765 (2022).

37. Rutherford, G. W. & Wlodarczyk, R. C. Distant Sequelae of Traumatic Brain Injury: Premature Mortality and Intracranial Neoplasms. *J. Head Trauma Rehabil.* **24**, 468 (2009).

38. Sabol, J., Kane, C., Wilhelm, M. P., Reneker, J. C. & Donaldson, M. B. The Comparative Mental Health Responses Between Post-Musculoskeletal Injury and Post-Concussive Injury Among Collegiate Athletes: A Systematic Review. *Int. J. Sports Phys. Ther.* **16**, 1–11.

39. Sui, S., Sun, J., Chen, X. & Fan, F. Risk of Epilepsy Following Traumatic Brain Injury: A Systematic Review and Meta-analysis. *J. Head Trauma Rehabil.* **38**, E289 (2023).

40. Tai, K. *et al.* Olfactory Dysfunction Following Moderate to Severe Traumatic Brain Injury: A Systematic Review and Meta-Analysis. *Neuropsychol. Rev.* (2022) doi:10.1007/s11065-022-09563-2.

41. Van Praag, D. L. G., Cnossen, M. C., Polinder, S., Wilson, L. & Maas, A. I. R. Post-Traumatic Stress Disorder after Civilian Traumatic Brain Injury: A Systematic Review and Meta-Analysis of Prevalence Rates. *J. Neurotrauma* **36**, 3220–3232 (2019).
42. Warren, S. A. *et al.* Traumatic Injury and Multiple Sclerosis: A Systematic Review and Meta-Analysis. *Can. J. Neurol. Sci.* **40**, 168–176 (2013).
43. Yau, K.-C., Revill, G., Blackman, G., Shaikh, M. & Bell, V. Pediatric traumatic brain injury as a risk factor for psychosis and psychotic symptoms: a systematic review and meta-analysis. *Psychol. Med.* **54**, 32–40 (2024).
