# Supplementary material for: An umbrella review of health outcomes following traumatic brain injury
Source: Nat Ment Health. 2025 Jan 3;3(1):83–91. doi: 10.1038/s44220-024-00356-5 (PMC11717692; doi:10.1038/s44220-024-00356-5)
Supplement: Supplementary file 2 — Reporting Summary [file 44220_2024_356_MOESM2_ESM.pdf]

nature portfolio

Corresponding author(s): Prof Seena Fazel

Last updated by author(s): Oct 14, 2024

Reporting Summary

Nature Portfolio wishes to improve the reproducibility of the work that we publish. This form provides structure for consistency and transparency in reporting. For further information on Nature Portfolio policies, see our Editorial Policies and the Editorial Policy Checklist.

Statistics

For all statistical analyses, confirm that the following items are present in the figure legend, table legend, main text, or Methods section.

n/a	Confirmed	
☐	☒	The exact sample size (*n*) for each experimental group/condition, given as a discrete number and unit of measurement
☐	☒	A statement on whether measurements were taken from distinct samples or whether the same sample was measured repeatedly
☐	☒	The statistical test(s) used AND whether they are one- or two-sided<br>*Only common tests should be described solely by name; describe more complex techniques in the Methods section.*
☒	☐	A description of all covariates tested
☒	☐	A description of any assumptions or corrections, such as tests of normality and adjustment for multiple comparisons
☐	☒	A full description of the statistical parameters including central tendency (e.g. means) or other basic estimates (e.g. regression coefficient) AND variation (e.g. standard deviation) or associated estimates of uncertainty (e.g. confidence intervals)
☒	☐	For null hypothesis testing, the test statistic (e.g. *F*, *t*, *r*) with confidence intervals, effect sizes, degrees of freedom and *P* value noted<br>*Give P values as exact values whenever suitable.*
☒	☐	For Bayesian analysis, information on the choice of priors and Markov chain Monte Carlo settings
☒	☐	For hierarchical and complex designs, identification of the appropriate level for tests and full reporting of outcomes
☐	☒	Estimates of effect sizes (e.g. Cohen's *d*, Pearson's *r*), indicating how they were calculated

Our web collection on statistics for biologists contains articles on many of the points above.

Software and code

Policy information about availability of computer code

Data collection	No code was used to collect the data. Articles were managed using Endnote X9 and Rayyan software (Rayyan is a SaaS and thus has no public version). The 'PRISMA2020' package was used to produce the PRISMA flow diagram.
Data analysis	Excel was used to manage the data. Risk estimates of health outcomes following TBI were visually displayed on a forest plot using the ‘metafor’ package in R Studio. 'ORToRelRisk’ from the ‘DescTools’ Package in R Studio was used to convert odds ratios (OR), and their confidence intervals, to RR. The ‘effectsize’ package was used to convert Cohen's d to OR. The Risk of Bias plot was created using the 'robvis' package.

For manuscripts utilizing custom algorithms or software that are central to the research but not yet described in published literature, software must be made available to editors and reviewers. We strongly encourage code deposition in a community repository (e.g. GitHub). See the Nature Portfolio guidelines for submitting code & software for further information.

Data

Policy information about availability of data

All manuscripts must include a data availability statement. This statement should provide the following information, where applicable:

- Accession codes, unique identifiers, or web links for publicly available datasets
- A description of any restrictions on data availability
- For clinical datasets or third party data, please ensure that the statement adheres to our policy

We did a keyword search on four major electronic databases via Ovid, Embase, MEDLINE, Global Health, and PsycINFO, alongside searching Cochrane Database of Systematic Reviews for papers published from database inception until 17 May 2023. All data was attained from published systematic reviews and meta-analysis and is cited in-text and in the reference list. The study protocol was published with PROSPERO (CRD42023432255) and is available at crd.york.ac.uk/PROSPERO/display_record.php?RecordID=432255.

Research involving human participants, their data, or biological material

Policy information about studies with human participants or human data. See also policy information about sex, gender (identity/presentation), and sexual orientation and race, ethnicity and racism.

Reporting on sex and gender	As an umbrella review of already published systematic reviews and meta-analysis, we were limited by the characteristics reported in the original reports. Information of sex was not consistently reported in the original articles.
Reporting on race, ethnicity, or other socially relevant groupings	Race, ethnicity and other socially relevant groups was not reported in the original articles.
Population characteristics	Individuals who have experienced traumatic brain injury (TBI) and individuals who had not experienced TBI (healthy controls and other injuries controls). Please see Methods and Table S3 for additional information.
Recruitment	New subjects were not recruited for this study. This study used already collected data.
Ethics oversight	n/a

Note that full information on the approval of the study protocol must also be provided in the manuscript.

Field-specific reporting

Please select the one below that is the best fit for your research. If you are not sure, read the appropriate sections before making your selection.

☒ Life sciences ☐ Behavioural & social sciences ☐ Ecological, evolutionary & environmental sciences

For a reference copy of the document with all sections, see nature.com/documents/nr-reporting-summary-flat.pdf

Life sciences study design

All studies must disclose on these points even when the disclosure is negative.

Sample size	The systematic review included a total of 31,397, 958 individuals exposed and unexposed to TBI, with participant numbers in each primary study ranging from 338 to 9,861,153
Data exclusions	Due to differences in pathological mechanisms, brain injury caused by hypoxia-ischemia was excluded. Non-systematic reviews, interventional studies, case studies, observational studies, discussion papers, editorials, letters, and conference abstracts were also excluded. Cognitive and social outcomes were considered outside scope. Disability measures such as return to work post-injury, were considered related to social outcomes and thus outside the scope of the current review.
Replication	We examined prediction intervals where possible, which indicate whether study findings could be replicated in future studies
Randomization	Randomization is not relevant due to the systematic review feature of the study.
Blinding	Blinding is not relevant as this is a systematic review

Reporting for specific materials, systems and methods

We require information from authors about some types of materials, experimental systems and methods used in many studies. Here, indicate whether each material, system or method listed is relevant to your study. If you are not sure if a list item applies to your research, read the appropriate section before selecting a response.

Materials & experimental systems

n/a	Involved in the study
☒	☐ Antibodies
☒	☐ Eukaryotic cell lines
☒	☐ Palaeontology and archaeology
☒	☐ Animals and other organisms
☒	☐ Clinical data
☒	☐ Dual use research of concern
☒	☐ Plants

Methods

n/a	Involved in the study
☒	☐ ChIP-seq
☒	☐ Flow cytometry
☒	☐ MRI-based neuroimaging

Plants

Seed stocks	*Report on the source of all seed stocks or other plant material used. If applicable, state the seed stock centre and catalogue number. If plant specimens were collected from the field, describe the collection location, date and sampling procedures.*
Novel plant genotypes	*Describe the methods by which all novel plant genotypes were produced. This includes those generated by transgenic approaches, gene editing, chemical/radiation-based mutagenesis and hybridization. For transgenic lines, describe the transformation method, the number of independent lines analyzed and the generation upon which experiments were performed. For gene-edited lines, describe the editor used, the endogenous sequence targeted for editing, the targeting guide RNA sequence (if applicable) and how the editor was applied.*
Authentication	*Describe any authentication procedures for each seed stock used or novel genotype generated. Describe any experiments used to assess the effect of a mutation and, where applicable, how potential secondary effects (e.g. second site T-DNA insertions, mosiacism, off-target gene editing) were examined.*
